# CD34+/CD38- Stem Cell Burden Could Predict Chronic Myeloid Leukemia Patients’ Outcome

**DOI:** 10.31557/APJCP.2021.22.10.3237

**Published:** 2021-10

**Authors:** Noura Fathy El-Metwaly, Salah Aref, Mohamed Ayed, Manal Abdel Hamid, Ahmed M.A. El-Sokkary

**Affiliations:** 1 *Biochemistry Subdivision, Department of Chemistry, Faculty of Science; Mansoura University, Mansoura, Egypt. *; 2 *Hematology Unit, Department of Clinical Pathology, Mansoura University, Mansoura, Egypt. *; 3 *Department of Medical Oncology, Mansoura University Oncology Center (MUOC), Mansoura, Egypt. *

**Keywords:** CML, CD34+/CD38, stem cells, outcome

## Abstract

**Background:**

The current predictor of the Chronic myeloid leukemia (CML) patients’ outcome is the degree of response to targeted therapy; here we search for a biomarker predicting CML outcome before start of therapy. This study aimed to assess the impact of the CD34+/CD38- stem cells (SCs) burden in chronic myeloid leukemia (CML) on treatment response and patients’ outcomes.

**Methods:**

Our study included 65 CML patients in the chronic phase. The patients’ CD34+/CD38- stem cells were quantified using flowcytometry before and after treatment by frontline imatinib (IM) therapy. The median follow-up for all patients was 18 months.

**Results:**

CD34+/CD38- stem cells frequency at diagnosis and after therapies are correlated to known prognostic markers (blast cells count, spleen size, total White cell count, and clinical scores). After therapy, the leukemic stem cells count dropped rapidly. The pretreatment CD34+/CD38- stem cells burden predicts response to frontline therapy. In addition, high SCs frequency at diagnosis predicts poor molecular response, transformation to AML, and poor patients’ outcomes.

**Conclusion:**

The percentage of CD34+/CD38- SCs burden at diagnosis reflects the CML disease behavior and is considered a biomarker for predicting CML patients’ response to first-line Tyrosine kinase inhibitors (TKI) therapy.

## Introduction

Chronic myeloid leukemia (CML) is a myeloproliferative neoplasm characterized by trilineage hyperplasia and arising at the level of a pluripotent stem cell. The prominent feature of CML is the presence of the BCR-ABL fusion gene (Down et al., 1964). The annual incidence of CML worldwide is 1-2 cases per 100,000 population, with a slight male predominance. The annual incidence increases with age, from < 0.1 cases per 100,000 children to > 2.5 cases per 100,000 elderly individuals. Due to the success of tyrosine kinase inhibitor (TKI) therapy in reducing mortality rates (down to only 2-3% per year), the prevalence of CML is expected to increase considerably (Swerdlow et al., 2017).

The Imatinib (IM) discovery in the early 21st century revolutionized the management as it induces morphologic, cytogenetic, and even molecular responses in the vast majority of patients. However, IM cannot remove all the leukemic stem cells (LSCs); the residual leukemic stem cells are the source of disease relapse after complete remission (Valent, 2008). Moreover, the second generation of TKIs, namely nilotinib and dasatinib, cannot eradicate all LSCs (Samanta et al., 2010). The CML disease resistance is classified into primary resistance or secondary resistance. The primary resistance is defined by the failure to achieve a hematologic or cytogenetic response. Hematologic resistance occurs in 2–4% of cases, while cytogenetic resistance is more common, occurring in 15–25% of patients (Elias et al., 2011). The primary resistance on a rare occasion is attributed to BCR-ABL gene mutations. Other causes of resistance are genetic amplification and expression of BCR-ABL or genetic aberration in leukemogenic signalling pathways and the remaining residual LSCs (Elias et al., 2011).

Although in vitro studies reported that most of the leukemic stem cells are responsive to TKIs, they cannot remove most of the dormant LSCs (Graham et al., 2002; Chomel et al., 2016). The in vivo resistance might be due to the overproduction of IL3, G/GM-CSF, which may offer more protection against IM and thereby to reduced cell killing (Jiang et al., 2007). The ability of the LSCs to persist after therapy forming a reservoir for relapse, disease progression, and resistance (Houshmand et al., 2019). 

CML relapse was observed in 50% of the patients who achieved deep genetic response after discontinuation of Imatinib during the first six months (Ross et al., 2013; Zhang et al., 2010). The previous studies that have tried to quantify bone marrow LSCs resulted in discordant findings; this is due to technical difficulties’ or the small number of studied cases (Thielen et al., 2016). Flowcytometric approaches have been suggested to discriminate normal from leukemic stem cells using a panel including CD45+/CD34+/CD38− in gating strategy (Bocchia et al., 2018; Herrmann et al., 2020; Kinstrie et al., 2020). 

The current study aimed to address CD34+/CD38- stem cells burden before and after treatment on the CML prognosis. 

## Materials and Methods

*Patients*

The present study was conducted on 65 newly diagnosed CML patients (27 females and 38 males) in the chronic phase before starting therapy. The patients’ age ranges from 17 to 83 years old (mean age = 43.83 years). The included patients were treated by Imatinib and evaluated every three months for evidence of hematological and molecular response. This study span from March 2016 to November 2019. The diagnosis was made according to WHO. We used flow cytometry to assess CD34+/CD38- stem cells burden in patient samples at diagnosis and molecular remission. CML patients were treated frontline therapy, namely Imatinib. The median follow-up for all patients was for 18 months. The patients who did not achieve deep molecular response were treated by second-line therapy. 

*Inclusion criteria*

• Newly diagnosed CML patients with no treatment.

• The patients should have been in chronic phase CML.

• Written informed consent before any study procedures being performed.

*Exclusion criteria*

• Patients with history of exposure to chemotherapy/radiotherapy.

• Patients who were in accelerated phase or blastic crises at diagnosis or those who progressed to either phases during the course of the study.

• Cases of CML that have different specific protocol of treatment.

*Patients were subjected to the following clinical and laboratory studies*

1. Detailed history taking: including name, age, sex, residence and bone aches.

2. Clinical examination: general and local examination including examination of liver, spleen, and lymph nodes.

3. Abdominal ultrasonography: to demonstrate the presence of hepatomegaly or splenomegaly.

4. Clinical risk scores : as Sokal score, Hasford score, EUTOS score and ELTS score.

5. Laboratory investigations: a complete data sheet was taken includes investigations for diagnosis of CML.

*Quantification of SCs by flow-cytometry*

To quantify SCs (CD34+/CD38-), we used flow-cytometry based on stain/lysis/washing maneuver. In one tube, 10µl fluorescein isothiocyanate (FITC) anti-human CD34 (code: 343504), 10µl phycoerythrin (PE) anti-human CD38 (code 303506) purchased from Bio Legend, USA and 100µl of EDTA bone marrow (BM) fresh samples were added. After that, the tube content was mixed and incubated at room temperature for 15 minutes. After that, the cells were washed twice with phosphate-buffered saline (PBS); 2ml lysis buffer (cat no:S3325,Dako), was added, mixed by vortex, and incubated for 15 minutes in the dark, and again the cells were washed twice with PBS. After the last wash, the cells were suspended in 1ml of PBS, mixed well by vortex, and then analyzed using a flow cytometer (BD FACS Canto ™II Flow Cytometer with FACS Diva software: Becton Dickinson, USA). At least 100,000 events/tube were measured. 

The SCs in fresh samples were gated using CD34 by forwarding Scatter (FSC) and Side Scatter (SSC) light properties; then, sequential gating was carried out on CD34+/CD38− subpopulation. 

*Statistical analysis*

This study’s primary objective is to address whether the value of the biomarker CD34+CD38- SCs in the diagnosis phase can predict the patient’s response to TKI therapy and the follow-up the transformation into AML, and prediction of death. The analyses were done by the SPSS program (Standard version 24). 

The normality of the obtained data was first tested using the Kolmogorov-Smirnov test applied to one sample. Continuous variables were presented as mean ± SD (standard deviation) for parametric data and median (min-max) for non-parametric data. Qualitative data were described using number and percent. The association between categorical variables was checked using the Chi-square test. Fischer’s exact test was used if the cell count is less than 5. The comparison between the two groups was made using the t-test for parametric data and the Mann-Whitney test for non-parametric data. The paired groups were compared by paired t-test (parametric) and Wilcoxon signed-rank test (no parametric). Cox regression analysis was done using prognostic variables on univariate analysis, and the significant parameters were introduced in multivariate analysis for the prediction of death. A logistic regression model was used to predict response and transformation for entering statistical technique to predict the most significant determinants and control for possible interactions and confounding effects. For all analyses, P values <0.05 were considered significant. Kaplan- Meier test was done for survival analysis, and statistical significance of differences among curves was determined by the Log-Rank test. 

## Results

*Demographic Data and Patients’ characteristics*

The clinical and laboratory findings in the studied CML patients’ group are shown in Table 1. This study was conducted on 65 patients who were newly diagnosed with chronic myelogenous leukemia (CML); all are in the chronic phase. The group is formed of 27 females and 38 males. the patients’ age ranges from 17 to 83 years old (average age of 43.83 years), we used flowcytometry to quantify CD34+/CD38- stem cells in patient samples; all samples contain CD34+CD38- with variable ranges .patients treated frontline with imatinib. All patients have different demographic data as shown in Table 1. Median follow-up for all patients was at least 18 months. 

*CML laboratory data at diagnosis versus at those at six months post-therapy*

The recorded laboratory findings before and after imatinib therapy, namely hemoglobin levels; total WBCs counts; Platelets count; BM cellularity and BM blast cells %, BCR-ABL%, SCs burden; serum Uric acid; serum creatinine; revealed that there is a significant reduction in TLC, BCR-ABL %, BM cellularity, and SCs burden (Table 2). Laboratory investigation at diagnosis shows that there is high proportion of SCs (CD34+CD38-) in patients with high leukocyte count, enlarged spleen size. Similarly, patients with high SCs count at diagnosis had more often low red blood cells count, low hemoglobin, high platelet count, high HDL, higher kidney and liver function tests and high BCR-ABL. The proportion of SCs (CD34+CD38-) after treatment was low in patients with low leukocyte count, high red blood cells ,high hemoglobin, low platelet count ,low HDL, lower kidney and liver function tests and low BCR-ABL

*Correlations between CD34+CD38- stem cells burden and Prognostic markers*

The frequency of leukemic stem cells at diagnosis and after imatinib therapy showed a significant correlation with clinical scores; splenomegaly; Blast cells count; BCR-ABL %; LDH levels (Table 3). 

*Predictive value of CD34+CD38- burden on response to frontline TKI Therapy*

To predict response to therapy, logistic regression was used using univariate analysis and multivariate analysis. The multivariate analysis revealed that the leukemic stem cell burden at diagnosis is a significant predictor of deep molecular response Odds ratio (OR) 31 (CI: 3.5-85) and post-therapy OR 9.6 (CI: 2.1- 43) (Table 4).

*CD34+CD38- predictive value of transformation of CML to AML*

To predict the transformation into another disease, logistic regression was applied using Univariate analysis and multivariate analysis; If CD34+CD38- is ˃0.25 before the treatment, the patient will be transformed to another disease as it is less than that the patient is in chronic phase CML; after the treatment; If CD34+CD38-is ˃0.09, the patient is transformed to AML; If less, the patient was still in the same phase of CML, and this allowed us to consider CD34+CD38- as a diagnostic tool to predict transformed patients (Table 5).

*Cox regression analysis to predict mortality*

Multivariate analysis to predict CML patients’ deaths was assessed using the following parameters: age, Sokal score, leukemic stem cell burden, and other laboratory data. The significant parameter in univariate analysis was introduced in multivariate analysis. These analyses revealed that CD34+CD38- SCs burden either at diagnosis or after treatment is a significant predictor of CML patient’s outcome. At diagnosis the Hazard ratio is 3.5 (CI: 1.2-18.2) and after therapy Hazard ratio 7.6 (CI: 2.7-35) (Table 6).

*The Kaplan-Meier Curves of CD34+CD38-&BCR-ABL of OS, PFS, and DFS*

The CML patient survival analysis was evaluated using the Kaplan-Meier curve (Figure 1a;1b;1c). The CML patient’s subgroup that harbored high CD34+CD38- SCs burden either before the start of therapy or at a major molecular response showed shorter OS; and shorter PFS. 

**Table 1:** CML Patients’ Characteristics

Patients	Parameters	
Age at Diagnosis	Mean ± SD	43.83±16.02
	Min-Max	17.00-83.00
Gender	Male	38 (58.5%)
	Female	27 (41.5%)
Splenomegaly	Average	9 (13.8%)
	Intermediate	20 (30.8%)
	Enlarged	16 (24.6%)
	Huge	20 (30.8%)
	Size /cm (Mean ± SD)	17.52±3.64
Hepatomegaly	Average	30 (46.2%)
	Intermediate	16 (24.6%)
	Enlarged	19 (29.2%)
Hepatitis B	Positive Hepatitis B	1 (1.5%)
	Negative Hepatitis B	64 (98.5%)
Hepatitis C	Positive Hepatitis C	14 (21.5%)
	Negative Hepatitis C	51 (78.5%)
Mortality	Died	13 (20.0%)
	Survived	52 (80.0%)
OS months	Median (Min-Max)	18.00 (12-42)
PFS months	Median (Min-Max)	18.00 (3-42)
	Remission	38 (58.5%)
Recurrence	Relapse	4 (6.2%)
	No response	23 (35.4%)
	Responder	38 (58.5%)
Response	Non responder	27 (41.5%)

**Table 2:** Laboratory Investigations before and after (3 Months) TKI Therapy among the Studied Group

Laboratory parameters	Before treatment (n=65)	After treatment (n=65)	P value
TLC Median (Min-Max)	179 (62-605)	6.80 (2.40-301)	<0.001*
HB (g/dL) Mean ± SD	10.59±2.21	11.31±2.38	0.048*
PLT (X10^3^/cmm) Median (Min-Max)	305 (39-1023)	172 (6-833)	<0.001*
BM Cellularity Mean ± SD (10^3^/cmm)	83.81±16.91	71.6±23.26	0.035*
BM blast % Median (Min-Max)	2.00 (1-10)	2.00 (1-85)	0.12
BCR-ABL% Median (Min-Max)	74.37 (4-347)	0.20 (0-149.5)	<0.001*
CD34+38- % Median (Min-Max)	0.25 (0.07-0.93)	0.09 (0.03-1.31)	0.026*
Creatinine Mean ± SD	1.09±0.34	1.07±0.42	0.63
Uric acid Mean ± SD	7.54±2.73	5.25±1.92	<0.001*

*significant p <0.05, paired t-test and Wilcoxon signed-rank tests were used

**Table 3:** Correlation between Stem Cells (CD34+/CD38-) Burden and Other Prognostic Factors

Parameters	CD34+38- stem cells burden at diagnosis		CD34+38-stem cells burden after treatment (6 months)	
	R- value	P - value	R - value	P - value
Sokal Score	0.43	0.001*	0.401	0.002*
Hasford Score	0.341	0.005*	0.447	˂0.001*
ELTS Score	0.298	0.016	0.336	0.012
EUTOS Score	0.291	0.019	0.407	0.002
BM Blast cells%	0.409	0.002	0.316	0.175
TLC×10^3^/cmm	0.261	0.036	0.379	0.004
BCR-ABL%	0.081	0.522	0.784	0.001
Splenomegaly	0.427	0.001	0.367	0.006
Initial LDH	0.333	0.007	0.471	0.001

Correlation is significant at p˂0.05 level; Spearman’s correlation Coefficient was used

**Table 4:** Logistic Regression Analysis to Predict Molecular Response

Parameters	Univariate Logistic regression	Multivariate Logistic regression
	OR (95%CI)	OR (95%CI)
Sokal Score Low /intermediate vs high	11 (2.8-42)	9.0 (2.1-38)
TLC (before) < vs≥ 179	3.4 (1.2-9.6)	1.5 (0.3-5.6)
TLC (after) ≤ vs>6.8	4.3 (1.5-12.4)	3.1 (0.9-11)
GOT (after) < vs≥25	3.8 (1.3-10.9)	8.2 (1.5-45)
BCR-ABL (after) < vs≥ 0.2	18.5 (5-67)	26 (1.8-89)
CD34+38- % (before) < vs≥ 0.25	56 (6.8-89)	31 (3.5-85)
CD34+38- % (after) ≥0.09 vs<	18.5 (5.1-67)	9.6 (2.1-43)
Initial LDH >vs≤1102	5.1 (1.7-15)	4.5 (1.2-16)

OR, odds ratio; CI, confidence interval; *, significant

**Table 5:** Logistic Regression Analysis to Predict Transformation of CML to AML

Parameters	Univariate regression	Multivariate regression
	OR (95%CI)	OR (95%CI)
Hasford risk (High)	11.4 (1.2-40.5)	2.1 (0.5-8.4)
TLC (after)	1.02 (1.01-1.03)	1.01 (0.9-1.03)
Hb (after)	1.7 (0.6-10.6)	-
Basopils	1.5 (1.1-2.3)	1.6 (0.8-1.02)
Eosinopils	1.3 (1.01-1.6)	1.1 (0.7-1.7)
Initial LDH	1.1 (1.001-1.5)	0.9 (0.6-61.9)
BCR-ABL (after)	1.05 (1.02-1.1)	0.95 (0.9-1.01)
CD34+38- % (before)>vs≤0.25	24 (11.5-54)	4.9 (1.57-69)
CD34+38- % (after) >vs≤0.09	12.4 (5.1-73)	2.7 (1.1-17.4)

OR, odds ratio; CI, confidence interval; *, significant

**Table 6:** Cox Regression Analysis to Predict Mortality

Parameters	Univariate Cox regression HR (95%CI)	Multivariate Cox regression HR (95%CI)
Age at diagnosis (>40 y)	5.5 (1.1-27.2)	1.78 (0.07-8.5)
Sokal Score (High)	10.3 (1.2-84)	2.83 (2.8-46)
Hb (after) >vs≤11.3	1.47 (0.3-1.7)	1.1 (0.3-1.4)
PLT (before) <vs≤305	2.6 (0.7-9.6)	-
PLT (after) <vs≤172	7.5 (1.5-37)	3.5 (0.6-20)
BCR-ABL (before) >vs≤74	10.9 (2.6-45)	9.1 (1.8-46)
CD34+38- % (before)>vs≤0.25	5.7 (2.4-28)	3.5 (1.2-18.2)
CD34+38- % (after) >vs≤0.09	8.1 (1.6-40)	7.6 (2.7-35)
Initial LDH >vs≤1102	2.8 (0.8-10)	1.5 (0.4-6)
TLC (after) >vs≤6.8	9.5 (1.9-47)	8.9 (1.7-38)

OR, odds ratio; CI, confidence interval; *, significant

**Figure (1a):**
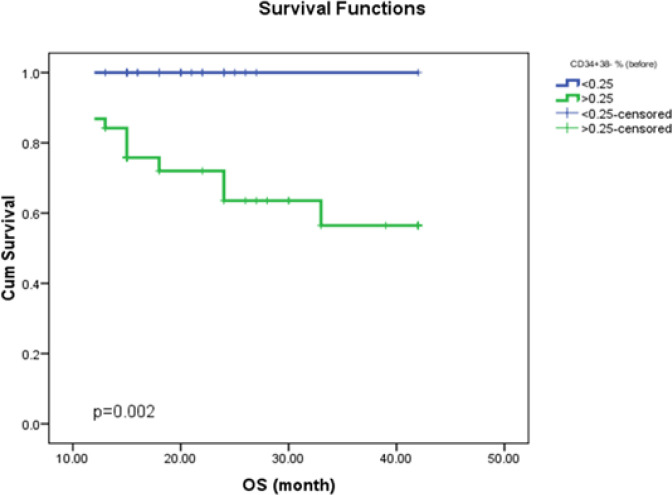
Impact of Stem Cell Burden (CD34+/CD38–) at Diagnosis on CML Patients Overall Survival. The analysis revealed that CML patients with high Stem cells burden have shorter overall survival time as compared with those with low SCs burden (P=0.002)

**Figure (1b):**
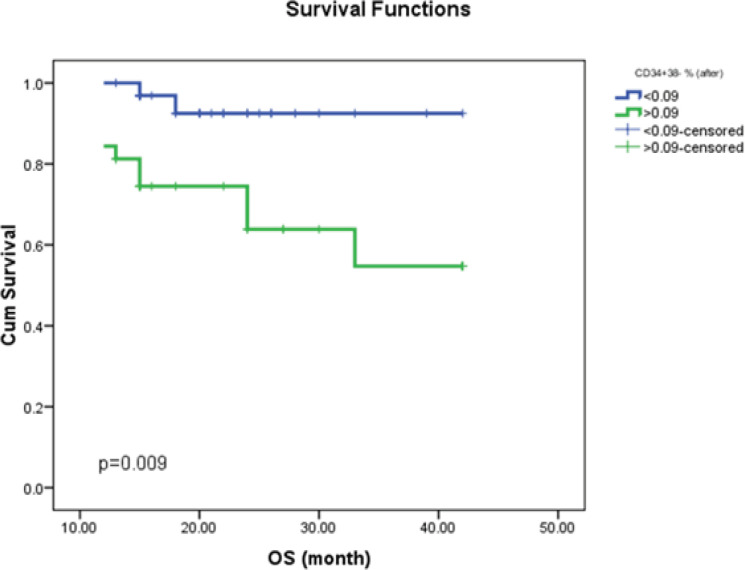
Impact of Stem Cell Burden (CD34+/CD38–) after Induction on Overall Survival in CML Patients. The analysis revealed that CML patients who harbored high Stem cell residue after three months of chemotherapy have shorter OS time as compared to those who harbored a low burden of Stem cells (P˂0.009)

**Figure (1c):**
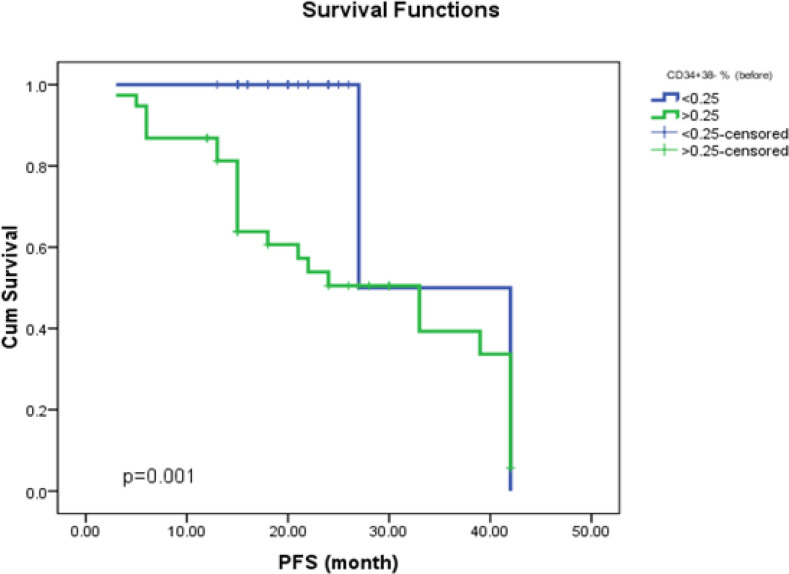
Impact of Stem Cells Burden (CD34+/CD38–) at Diagnosis on Progression-Free Survival in CML Patients. CML patients with low Stem cells burden had longer time of progression-free survival as compared with those who harbored a high Stem cell burden (P=0.001)

## Discussion

In this study, we addressed whether the quantification of stem cells frequency at diagnosis and after the start of therapy could predict the patient’s response to the first-line therapy and the CML patient’s outcome. 

Previous studies showed that the CD34+CD38- SCs quantification could be accurately quantified before and after the start of targeted therapy, which might have important implications for both effectiveness of different targeted therapy to eradicate CD34+CD38- SCs and the decision to therapy continuation (Chu et al., 2011).

Despite the prominent success of TKI in controlling the CML chronic phase, a high number of CML patients still develop drug resistance or relapsed after molecular response; these could be attributed to the failure of TKIs to eradicate all CD34+CD38- SCs, which are responsible for CML relapse (Zhou and Xu, 2015). 

In the current study, there is a significant reduction in WBCs counts; BCR-ABL%; total BM counts; serum Uric acid after the start of imatinib therapy. These findings are similar to those that were reported in previous studies (Thielen et al., 2016). 

The percentage of CD34+CD38- stem cells at diagnosis was correlated significantly to clinical scores, WBCs count, BM blast cells %, splenomegaly, and BCR-ABL %. These findings parallel with that reported by previous studies that assessed LSC by flow cytometry (Thielen et al., 2016) or that done by other techniques (Janssen et al., 2012; Mustjoki et al., 2013). Likewise, one previous study (Mustjoki et al., 2013) showed that the frequency of LSCs in chronic phase CML patients at diagnosis is highly variable and is considered a good prognostic biomarker that correlated with the other biomarkers, including WBCs count, percentage of blast cells as well as the degree of splenomegaly. 

Our results denoted that multivariate regression analysis revealed that CD34+CD38- SCs burden at diagnosis could significantly predict deep molecular response and good response to therapy. In the first two years, the patients responding to IM therapy showed a low frequency of progression to a more advanced phase (Jabbour et al., 2013). However, primary resistance to Imatinib occurs in a small number of patients, while others express primary response and then become resistant (secondary resistance) (Chereda and Melo, 2015). 

Genomic instability has been described in CML- chronic phase (CP) and most likely occurs in the LSCs enriched CD34+CD38– population and/or the Leukemic progenitor cell-rich CD34+ population. Therefore, CML- CP can progress to myeloid or lymphoid and sometimes biphenotypic acute leukemia (Chu et al., 2011; Valent, 2008). 

Multivariate regression analysis revealed that CD34+CD38- SCs burden at diagnosis could significantly predict a higher tendency to AML transformation. The statistical analysis results using univariate analysis and multivariate analysis revealed that higher CD34+CD38- SCs burden at diagnosis was significantly higher in cases transformed from chronic phase to accelerated phase, and dead patients compared to living ones. The previous reports stated the cause of progression in CML cases is that LSCs acquired novel additional genetic or epigenetic mutations (Janssen et al., 2012). 

Multivariate regression analysis revealed that CD34+CD38- SCs burden at diagnosis could significantly predict deep molecular response. The results showed that the lower CD34+CD38- SCs burden was associated with better overall survival. This finding paralleled previous studies that reported that the LSCs at diagnosis could be a good predictor of deep genetic response, OS and PFS (Mustjoki et al., 2013). 

In conclusion: The percentage of CD34+CD38- SCs burden at diagnosis reflects the CML disease’s behavior and is considered a biomarker for predicting CML patients’ response to first-line Tyrosine kinase inhibitors (TKI) therapy.

## Author Contribution Statement

Salah Aref: Conception and supervision. Noura Fathy El-Metwaly: Interpretation and analysis of the data. Mohamed Ayed: Laboratory work. Manal Abdel Hamid: Interpretation or analysis of data. Ahmed M.A. El-Sokkary, Preparation of the manuscript; Revision for Important intellectual. All authors wrote, revised and approved the manuscript.
